# Prevalence of Infection with Porcine Circovirus Types 2 and 3 in the Wild Boar Population in the Campania Region (Southern Italy)

**DOI:** 10.3390/ani11113215

**Published:** 2021-11-10

**Authors:** Maria Grazia Amoroso, Francesco Serra, Claudia Esposito, Nicola D’Alessio, Gianmarco Ferrara, Barbara Cioffi, Antonietta Anzalone, Ugo Pagnini, Esterina De Carlo, Giovanna Fusco, Serena Montagnaro

**Affiliations:** 1 Department of Animal Health, Istituto Zooprofilattico Sperimentale del Mezzogiorno, Portici, 80055 Naples, Italy; francesco.serra@izsmportici.it (F.S.); claudia.esposito.vet@gmail.com (C.E.); barbara.cioffi@izsmportici.it (B.C.); antonietta.anzalone@izsmportici.it (A.A.); direzionesanitaria@izsmportici.it (E.D.C.); giovanna.fusco@cert.izsmportici.it (G.F.); 2 Department of Veterinary Medicine and Animal Productions, University of Naples “Federico II”, 80137 Naples, Italy; jammaferrara@hotmail.it (G.F.); upagnini@unina.it (U.P.); semontag@unina.it (S.M.)

**Keywords:** porcine circovirus, PCV-2, PCV-3, wild boars, prevalence

## Abstract

**Simple Summary:**

A retrospective, large-scale molecular survey was carried out to detect the presence of the PCV-2 and PCV-3 genomes in wild boar samples in the Campania region. A total of 148 samples from wild boars were tested for PCV-2 and PCV-3 by real-time PCR. The combined prevalence was 74.32%. The percentage of coinfected animals was 22.30%.

**Abstract:**

In recent years, porcine circovirus (PCV) infection has been documented as an important and emerging health concern for livestock and wildlife worldwide. The purpose of the present study was to assess the molecular prevalence of PCV-2 and PCV-3 and to clarify the epidemiological role of wild boars in the circulation of this virus in Campania, Southern Italy. For this purpose, samples from several organs were collected during the hunting season 2017–2018 from 148 wild boars in the Campania region. Quantitative real-time PCR was used for the detection and quantification of PCV-2 and PCV-3 genomes. The combined prevalence of PCV-2 and PCV-3 was 74.32% in the wild boars tested. The proportions of wild boars positive for PCV-2 or PCV-3, or coinfected, were 47.30%, 49.32%, and 22.30%, respectively. No link was detected between PCV positivity and location, but gender was a risk factor for the disease (female; *p* < 0.0001; OR 0.29). Furthermore, our study provides a snapshot of PCV-2 and PCV-3 circulation in wild boars in the Campania region: our findings can help us to better understand the role of wildlife in PCV circulation.

## 1. Introduction

Porcine circoviruses (PCVs), genus Circovirus, family Circoviridae, are the smallest non-enveloped, single-stranded DNA viruses, characterized by a circular genome of approximately 2000 nt and icosahedral virions [1].

Currently, four species of PCV are known: porcine circovirus type 1 (PCV-1), type 2 (PCV-2), type 3 (PCV-3), and type 4 (PCV-4) [2,3,4,5]. PCV-1 was first detected in 1974 as a contaminant of PK-15 cell culture and is considered innocuous for swine [6]. PCV-2 was identified as an etiological agent of PCV-associated disease (PCVD) [7], which comprises postweaning multisystemic wasting syndrome (PMWS), pneumonia, porcine dermatitis, nephropathy syndrome (PDNS), and reproductive failure. However, it has been demonstrated that only some pigs show clinical signs of PCV-2 infection, whereas other swine show no clinical signs and maintain good performance [8]. It seems true that several factors, such as the viral titer, genetics, and immune response of the host, are involved in the genesis of clinical disease [9,10]. Usually, to cause disease, PCV-2 requires simultaneous infection with other microorganisms, such as porcine parvovirus (PPV), porcine reproductive and respiratory syndrome virus (PRRSV), and *Mycoplasma hyopneumoniae* [6]. PCV-2 infection can be subclinical, and it is considered endemic in several countries. in which it causes large economic losses in the pig industry.

PCV-3 was only recently discovered; it was first identified in the USA in 2017 and was subsequently detected in several countries such as Asia, South America, and Europe [11,12,13,14], but retrospective studies suggest that PCV-3 has been circulating among pigs for much longer [14].

PCV-4 was identified in 2019 in China [5], and has subsequently been detected in Korea [15] and Malaysia [16], but the virus was absent in swine samples from Europe (Spain and Italy) [17]. Currently, little is known about the pathogenesis of PCV-4, except that it is epidemiologically associated with respiratory, gastrointestinal, and PDNS diseases [5].

The European wild boar (*Sus scrofa*) is an omnivorous and polygamous species with an autumn reproductive season influenced by environmental conditions. Wild boars are native in many countries globally and are reservoirs for many diseases that are transmissible from animals to humans and vice versa [18]. The epidemiological role of wild boars as a reservoir and source of infection for humans and animals is still a controversial topic, as it depends on the disease and on the pathogen involved. For example, while for diseases such as tuberculosis, brucellosis [19,20], and African and classical swine fever, both the epidemiological status of wild animals and the interference in eradication plans are known [21]; this link is less evident for other diseases, such as porcine reproductive virus and respiratory syndrome (PRRSV) and PCV-2 [22]. Circoviruses are known to cause diseases in wild boar [23]; in particular, PCV-2 causes a characteristic lymphocyte depletion by affecting the immune system of the wild boar, producing immuno-suppression and exacerbation of concomitant infections such as tuberculosis [24]. Moreover, Risco et al. have shown that wild boar suffering from tuberculosis and coinfected with the PCV-2 species seems to have a greater likelihood of shedding M. tuberculosis DNA by nasal routes [25]. Consequently, the data about the prevalence in wildlife of infectious diseases, such as PCV, could be useful to better design control and eradication programs against infectious diseases of livestock and wild fauna.

Thanks to the ability of the boar to adapt to different environments, high fertility, and increased contact with man, in recent years, many European countries have experienced increases in wild boar populations in wooded and urban areas [26,27].

Consequently, the potential risk of transmission of infectious diseases among wild boar and domestic pigs is not negligible. In this regard, it should be remembered that PCV-2 and PCV-3 are widespread in the wild boar population without associated mortality [26,27,28,29].

Furthermore, PCV-2 has often been detected in flies (*M. domestica*) and culex mosquitoes in pig farms, and PCV-3 has been identified in wild ruminants and their related hematophagous ectoparasites (ticks) [30].

Therefore, the purposes of the present study were to establish, through a retrospective large-scale molecular survey, the prevalence of PCV-2 and PCV-3 in wild boars, and their quantitative tissue distributions; and to provide a snapshot of the circulation of these viruses in the Campania region.

## 2. Materials and Methods

### 2.1. Ethical Approval

Our study did not require ethical approval, since no live animals were used for this study. All samples were provided by hunters with appropriate wild boar hunting licenses, and animals were harvested by standard procedures for the hunting of wild boar. Harvested wild boars were carried to a central processing site for cleaning, dressing, and sampling procedures; and then, aliquots of organs were sent to Experimental Zooprophylactic Institute of Southern Italy for PCV investigation.

### 2.2. Study Area and Sample Site

Campania is in Southern Italy and overlooks the Tyrrhenian Sea. It covers an area of 13,595 km^2^ (41°00′00″ N–14°30′00″ E), with a characteristic temperate Mediterranean climate. 

The survey was conducted on 148 wild boars from 2 to 4 years of age. The animals were collected in Campania during the 2017–2018 hunting season within the Regional Plan for Wildlife Monitoring. For each animal, we recorded the location, gender, and date of collection.

The sample size was calculated using the formula proposed by Thrusfield et al. [31] for a theoretically “infinite” population. We added the following information: expected prevalence of PCV (20%) [32], confidence interval (CI) 95%, and desired absolute precision (0.07).

### 2.3. Materials

Samples from brain, heart, liver, and spleen were collected during the in-house slaughtering of the 148 hunted animals and sent to the Experimental Zooprophylactic Institute of Southern Italy for PCV analysis. Organs that arrived in bad condition were discharged. In total, 104 brains, 119 hearts, 136 livers, 120 and 126 spleens were analyzed. From a few animals, samples from other tissues were also collected—intestines (9), lungs (18), lymph nodes (1), and kidneys (1)—for a total of 514 analyzed samples.

### 2.4. Viral Nucleic Acid Extraction Procedures

For nucleic acid extraction, 25 mg of tissue was homogenated by Tissue Lyser (Qiagen GmbH, Hilden, Germany) in 2 mL Eppendorf safe-lock tubes containing 1 mL phosphate-buffered saline solution (PBS) and a 4.8 mm stainless steel bead (30 Hz for 5 min). Extraction was carried out on 200 µL of homogenate by using a QIAsymphony automated extraction system (Qiagen GmbH, Hilden, Germany) with the DSP Virus/Pathogen Mini kit (Qiagen GmbH, Hilden, Germany) according to the manufacturer’s instructions. Nucleic acids were eluted in 80 µL of elution buffer. A sample made with 200 µL of PBS instead of homogenate was used as a negative process control (NPC). PCR inhibitors likely present in the samples were monitored by adding an external process control (EPC), namely, murine norovirus [33], 5 µL of which (10^7^ PFUmL-1) waws spiked in each sample prior to extraction. EPC was amplified in each sample by real-time PCR with the following primers: MNoV F 5′-CACGCCACCGATCTGTTCTG-3′ and 5′-GCGCTGCGCCATCACTC-3′; and probe FAM-CGCTTTGGAACAATG-MGB-NFQ with the thermal profile indicated in the literature [34]. Results were analyzed as follows: if the threshold cycle (Ct) of the EPC in the eluted sample was comparable to that of the EPC in the NPC, the sample was analyzed as undiluted. If, instead, the difference between the two Cts was at least 3 or a multiple of 3, all the analyses were carried out on the sample diluted 1:10 or more (considering one decimal dilution every 3 threshold cycles of difference).

### 2.5. Real-Time PCR for the Differential Detection and Quantification of PCV-2 and PCV-3 

Circovirus was investigated in all the samples by a real-time quantitative PCR able to simultaneously identify and distinguish between porcine circovirus types 2 and 3 [35]. Differential detection was carried out on a QuantStudio 5 real-time PCR thermal cycler (Thermo Fisher Scientific, Waltham, MA, USA) in a total volume of 25 µL containing 5 µL of nucleic acids extract, 12.5 µL of TaqMan^®^ Universal PCR Mas-ter Mix (Thermo Fisher Scientific), and 1 µL each of the following primers (10 µM) and probes (5 µM): PCV-2-For (5′-CCAGGAGGGCGTTSTGACT-3′), PCV-2-Rev (5′-CGYTACCGYTGGAGAAGGAA-3′), PCV-3-For (5′-CGGTGGGGTCATATGTGTTG-3′), PCV-3-Rev (5′-CACAGCCGTTACTTCACC-3′), PCV-2-P (FAM-5′-AATGGCATCTTCAACACCCGCCTCT-3′-TAMRA), and PCV-3-P (VIC-5′-CTTTGTCCTGGGTGAGCGCTGGTAG-3′-BHQ2). The thermal profile was the following: initial denaturation for 15 min at 95 °C, 40 cycles of amplification for 10 s at 95 °C and for 30 s at 60 °C. Nuclease-free water was included as a negative control. Quantification for each target was carried out using a standard curve: analyzing serial dilutions of a standard and plotting the log of genome copies/µL (gc) against the Ct number. In detail, the PCV-2 standard curve was obtained by analyzing dilutions (from 1 × 10^10^ to 1 × 10^2^ copies/µL) of a standard obtained from VetMAX Porcine PCV-2 Quant kit (Thermo Fisher Scientific Inc., Waltham, MA USA). The PCV-3 standard curve was instead obtained by diluting (from 1 × 10^7^ to 1 × 10^1^ copies/µL) a plasmid [36] kindly given by the University of Padova. 

### 2.6. Statistical Analysis

Data analysis was performed using MedCalc Statistical Software version 20.015 (MedCalc Software, Ostend, Belgium; www.medcalc.org, accessed on 24 May 2021). Chi-square tests were used to compare proportions of positivity related to categorical dependent variables and to establish statistical significance within each class (gender and location).

The variables associated with seroprevalence for PCV were applied to binary logistic models using JMP Pro version 15.0.0 (SAS Institute Inc., Campus Drive, Cary, NC, USA). *p* < 0.05 was considered significant. Significant differences between categories were quantified by calculating odds ratios (OR) and their 95% confidence intervals (CI).

## 3. Results

### 3.1. PCV-2 and PCV-3 Detection

A total of 148 sampled wild boars were tested. Information about location was recorded for all samples; gender was recorded only for 138 animals. There were 76 males (51.3%), 62 females (42%), and 10 with undefined gender (6.7%). The province of Salerno provided the largest number of samples (50%, *n* = 74), followed by Caserta (22%, *n* = 32), Avellino (20%, *n* = 30), and Benevento (8%, *n* = 12).

**Figure 1:**
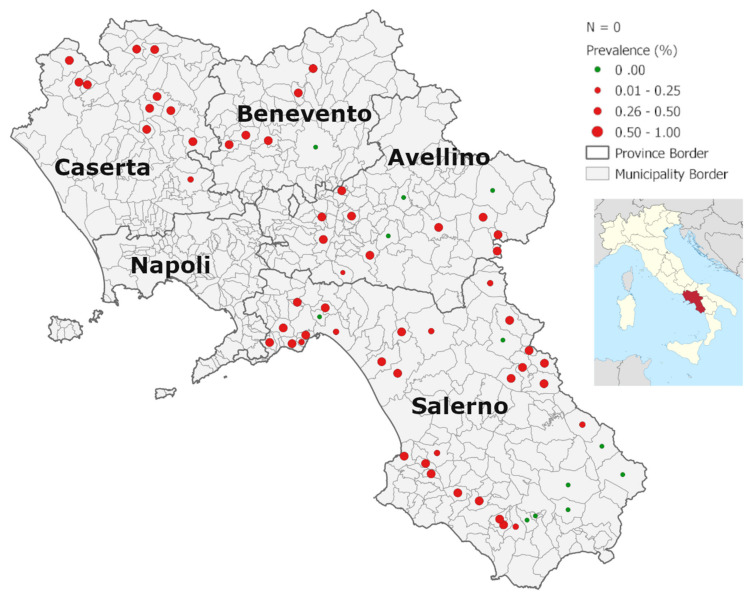
A map of the Campania (Italy) showing the distribution of samples of Eurasian wild boar (*Sus scrofa*) positive for porcine circoviruses. Apparent prevalences of PCV contact in the municipalities. Dot sizes are proportional to observed prevalences.

The combined prevalence of PCV-2 and PCV-3 was 74.32% (110/148, 95% CI: 67.29–81.36%) (Figure 1 and Table 1). In addition, the proportions of wild boars positive for PCV-2, PCV-3, or both were 47.30% (70/148, 95% CI: 39.25–55.34%), 49.32% (73/148, 95% CI: 41.27–57.38%), and 22.3% (40/148, 95% CI:15.59–29.0%), respectively (Table 1).

The genome of PCV was detected in a variable proportion of wild boars from all tested municipalities of the Campania region, ranging from 20 positive samples out of 30 (66.6%) in the province of Avellino to 29 out of 32 (90.6%) in the province of Caserta (Table 2).

The univariate and multivariate analyses showed a statistical association between gender (Chi-square test: DF = 1, *n* = 148, *p*-value = < 0.0001) and PCV positivity; in fact, a risk of PCV positivity was significantly correlated with female gender (91.94%, 95% CI 85.16–98.71), with an odds ratio of 8.29 (95% CI 2.98–23.02) (Table 2).

Data analysis indicated that there was no statistical association between PCV positivity and location (Chi-square test: DF = 3, *n* = 148, *p*-value = 0.1108); however, the highest percentage of positive animals was observed in Caserta province (90.6%, 95% CI 80.53–100.0) with odds ratios of 4.38 (95% CI 1.17–19.8), 3.22 (95% CI 0.55–18.84), and 4.09 (95% CI 1.12–14.8), respectively, versus Avellino, Benevento, and Salerno provinces (Table 2). PCV-2 and PCV-3 were both found in all the types of organs analyzed. 

The frequencies of detection of PCV-2 and PCV-3 were found to be comparable in the brain and heart, whereas in the liver and spleen, high PCV-2/PCV-3 frequencies were observed (liver 65.4%; spleen 72.2%). The total frequencies of detection of PCV (2 and 3) in the different tissue samples tested are shown in Table 3. Briefly, the PCV-2/PCV-3 genome was found in 57.7% (60/104, 95% CI: 48.2–67.2) of the brain samples (20.2% PCV-2, 20.2% PCV-3, and 17.3% coinfected); in 56.3% (67/119, 95% CI: 47.4–65.2) of heart samples (16.8% PCV-2, 21.8% PCV-3, and 17.6% coinfected); in 65.4% (95% CI: 57.5–73.49) of livers (22.1% PCV-2, 21.3% PCV-3, 22.1% PCV-2 and 3); and in 72.2% (95% CI: 64.4–80.04) of spleen samples (23.0% PCV-2, 24.6% PCV-3, 24.06% coinfected). PCV was also found in 61.1% (11/18, 95% CI: 38.59–83.63) of the lung samples tested (five PCV-2, two PCV-3, and four coinfected) and in 44.4% (4/9) of the intestines analyzed (three with PCV-2 and one with coinfection).

PCV was also found in 78.6% (11/14, 95% CI: 57.08–100.0) of the lung samples analyzed (five PCV-2, two PCV-3, and four with coinfection) and in 33.3% (3/9, 95% CI: 2.53–64.13) of the intestines analyzed (two with PCV-2 and one with coinfection). Furthermore, the only samples from the lymph nodes and kidney investigated were positive for both PCV-2 and 3.

### 3.2. PCV-2 and PCV-3 Quantification

Preliminary experiments were carried out to set up the standard curves for the quantification of the two PCVs. The PCV-2 standard curve equation was the following: y = −3.139x + 36.895, with an amplification efficiency (E) of 108.259% and an R^2^ = 0.998. The limit of quantification (LOQ) was 1 × 10^2^ copies/µL. The resulting PCV-3 standard curve equation was y = −2.494x + 28.795 with E = 151.737% and R^2^ 0.991. The LOQ for PCV-3 was 1 × 10^1^ copies/µL.

With the two standard curves (one for each of the two viral targets), all the samples were investigated for the quantitative presence of PCV-2 and PCV-3. Results were considered positive when Ct was <40. The amount of DNA per sample type ranged from 1.47 × 10^8^ copies/µL to values under the LOQ in spleen samples; from 4.28 × 10^5^ copies/µL to values under the LOQ in the liver; from 1.44 × 10^5^ copies/µL to values under the LOQ in the heart; and from 8.59 × 10^4^ copies/µL to values under the LOQ in the brain. With respect to single viruses (Table 4), the average PCV-2 concentration in the various organs was always higher than PCV-3. In detail, in the spleen, it was 4.98 × 10^6^ copies/µL for PCV-2 versus 2.0 × 10^3^ copies/µL for PCV-3; in the liver, it was 1.69 × 10^4^ copies/µL for PCV-2 versus 2 × 10^1^ copies/µL for PCV-3; in the heart, 7.66 × 10^3^ copies/µL for PCV-2 versus 1,5 × 10^1^ copies/µL for PCV-3; and in the brain, 4.98 × 10^3^ copies/µL for PCV-2 with respect to 1 × 10^1^ copies/µL for PCV-3.

## 4. Discussion

The study of PCV-2 and PCV-3 in the feral pig population is important to better understand the host’s susceptibility and its epidemiological significance in the spread of the disease. This research is the first study describing the presence of PCV-2 and PCV-3 in wild boars of the Campania region (Southern Italy).

Porcine circoviruses are ubiquitous in domestic and wild pig populations, as indicated in many studies [32,37,38]. Therefore, it was expected that PCV-2 and PCV-3 would be found in most of the wild boar samples analyzed. In fact, the PCV-2/PCV-3 genome was detected in 110 out of 148 analyzed samples, with an overall molecular prevalence of 74.32% (95% CI 67.29–81.36) and a coinfection rate of 22.3% (33/148, 95% CI: 15.59–29.0%) (Table 1). These values are higher than those reported by Saporiti et al. [32] in a study on detection by molecular methods of PCV-2 and PCV-3 in serum samples from fattening pigs of different European countries. In the above-named study, the overall prevalence was about 30% in Europe and about 20% (13/67) in Italy.

PCV-2 and PCV-3 co-infections have only been evaluated in domestic pigs in Korea [39], China [37], and in one study conducted in Europe [32]. The coinfection rate found in our study (22.3%, 33/148, 95% CI: 15.59–29.0%) in wild boars in the Campania region is lower than the value found in Korea (35.0%) [39] but is higher than the coinfection rate described in China (6.78%) [37]. Finally, our data disagree with the coinfection rate values described by Saporiti et al.—around 2.6% in Europe and around 1.5% in Italy [32].

The discrepancy between our results and those reported by Saporiti et al. [32] could be attributable to the nature of the sample used and the difference between the species (domestic swine and wild boars). As a confirmation of this, several studies conducted on domestic pigs showed that the detection of the circovirus genome is easier in tissues than in serum samples. In particular, the average viral load of PCV-2 in serum and tissue samples from domestic swine seems to depend on the stage of infection (PMWS or subclinical infection) [10,11,12,13,14,15,16,17,18,19,20,21,22,23,24,25,26,27,28,29,30,31,32,33,34,35,36,37,38]; additionally, the PCV-3 genome was detected by Klaumann et al. in wild boars, in all types of tissue tested, and in feces, with higher frequencies and viral loads than serum [38]. Therefore, the detection of the PCV genome in wild boar serum samples could be influenced by the stage of the disease and underestimate the prevalence of infection.

A further factor that can justify the discrepancies between our results and those of Saporiti et al. [32] is the application of good farm management practices (such as routine vaccination against PCV-2) for the control of infectious diseases; these are frequent in pig farming but absent for wild boar, and in general, for wild animals. Therefore, it stands to reason those different applications of good management practices may impact the prevalence values of infectious diseases in wild animals.

PCV-2 is an emerging pathogen distributed around the world, and it is believed to have a serious economic impact on the pig farming and swine industry in several countries [9].

In our study, PCV-2 molecular prevalence was 47.30% (95% CI: 39.25–55.34); this value is lower than the value reported by Dei Giudici et al. [40] in domestic and wild pigs in the Sardinia region (81.6%) but is higher than the value found in Italy (14.9%) by Saporiti et al. [32] in domestic pigs. In other European countries, PCV-2 infection seems to be as common as we observed in the Campania region; in fact, similar molecular prevalence values were found in Slovakia (from 43.8 to 49.0%) [41,42], in Germany (50.7%) [43], in Serbia (40.32%) [44], and in Portugal (44.6%) [45]. In other European countries, PCV-2 infection appears to be less common than we have observed in the Campania region; in fact, lower molecular prevalence values were found in Hungarian wild boars (20.5%) [46] and in fettering pigs in Belgium (29.0%) [32]. Finally, higher prevalence values were found in wild boars in Poland (75.6%) [47] and in Romania (Transylvania region) (100%) [48], and in domestic pigs in France (70.1%) [32].

Overall, 73 wild boars analyzed were PCV-3-positive, corresponding to a prevalence value of 49.32% (95% CI: 41.27–57.38%). This value is comparable with PCV-3 prevalence found in wild boars in the Friuli Venezia Giulia region (44.8%) [27], in the Sardinia region (61.54%) [40], and in Spain (42.66%) [49]. Significantly lower prevalence values were found in other Italian regions, such as the Veneto region (33.51%), and in domestic swine in Italy (34.4%) [50]. A significantly lower prevalence value was also found in other European countries, such as Germany (29.2%) and Slovakia (19.1%) [32].

In any case, it should be remembered that it is necessary to be cautious when comparing the prevalence values obtained in different geographical areas with different molecular tests and with different sampling methods; all these variables can influence prevalence value and over- or underestimate the impacts of infectious diseases on wildlife populations.

Univariate and multivariate analyses indicated that there was no correlation between location and molecular positivity to PCV-2 and PCV-3, even though a higher prevalence was found in animals living in the province of Caserta. The lack of association between molecular positivity and location is a surprising result. Probably, a larger sample would give more accurate statistical results, but this was not possible in our study. Nevertheless, possible explanations of this trend are a change in the composition of the wild boar population due to the hunting pressure, the expansion of the species, and the ethology of the wild boar [19]. Our data showed that the prevalence of infected animals was positively correlated with the female gender (*p* < 0.001). This result is probably since, in wild boars, contact between adult females and young animals is more likely than among male adults. In fact, wild boars are typically social animals, living in female-dominated sounders consisting of barren sows and mothers with young led by an old matriarch. Male boars leave their sounder at the age of 8–15 months, while females either remain with their mothers or establish new territories nearby. Subadult males may live in loosely knit groups, whereas adult and elderly males tend to be solitary outside of the breeding season [51].

When looking at the distributions of the two circoviruses in the samples investigated, PCV-2 and 3 were detected in all the types of organs analyzed, in accordance with previously published studies [37,38,39,40,41,42,43,44,45,46,47,48,49,50,51,52]. Both viruses showed the highest prevalence in the spleen (positivity in 48% of samples for PCV-2 and PCV-3), followed by the liver (44% of samples were positive for PCV-2 and 43% for PCV-3), confirming the tropism of the two viruses for these organs [37,38,39,40,41,42,43,44,45,46,47,48,49,50,51,52]. Furthermore, the higher viral loads of the circoviruses in the spleen (compared to the other organs) suggest that it could be a target for these viruses’ replication.

Our data showed very variable titers for both the circoviruses, ranging from <LOQ to 1.47 × 10^8^ copies/µL for PCV-2; and from <LOQ to 1.56 × 10^4^ copies/µL for PCV-3. PCV-3 never reached the high concentrations measured for PCV-2; furthermore, it was found in lower average amounts in all the types of organs analyzed (see Table 4). The literature has reported that high PCV-2 and PCV-3 viral loads are needed for systemic disease, whereas low viral loads are associated with subclinical disease or are found in healthy animals [7,8,9,10,11,12,13,14,15,16,17,18,19,20,21,22,23,24,25,26,27,28,29,30,31,32,33,34,35,36,37,38,39,40,41,42,43,44,45,46,47,48,49,50,51,52]. In detail, Segalés [7] reported concentrations of PCV-2 < 10^5^ to 10^6^ copies/mL of serum in subclinical infections, compared to concentrations of the virus >10^6^ copies/mL serum or >10^5^ per 500 ng of total DNA extracted from various organs in animals affected by systemic disease. On the same line, Alomar et al. 2021 [52] described, with respect to PCV-3, viral loads of around 1.5 × 10^3^ copies/mL in the organs of healthy pigs and values ranging from 1.2 to 9.38 × 10^7^ copies/mL in diseased pigs.

PCV-3 was widely distributed, but since it was found in low concentration in the samples, it should not be recognized as an etiology in PCVAD of wild boars. On the contrary, PCV-2 average viral loads were not neglectable and could indicate a pathological condition for the animals investigated. In this regard, we have no information about the states of health of the wild boars analyzed, since they were wild and were slaughtered by hunters for private consumption, and did not undergo any necropsy. Regardless of the state of health of the animal, however, high PCV-2 viral loads suggest that wild boars are a good reservoir of the virus and could represent a concrete risk of spread to domestic pigs.

## 5. Conclusions

This was the first molecular prevalence survey carried out on the wild boar population for PCV-2/PCV-3 in the Campania region. These results show that wild boars are susceptible to PCV-2 and PCV-3, which can influence the population’s dynamics (e.g., demographic increase) and its health. 

Furthermore, our study showed that PCV-2 and PCV-3 are widespread throughout the Campania region with a high rate of coinfection; this might indicate, in wild boars, the presence of a dependent circulation pattern for each virus. However, further studies on the direct effects of these pathogens on wild boars are necessary.

## Figures and Tables

**Table 1:** Molecular prevalences of PCV-2 and PCV-3 infections and co-infections in the wild boar population (*n* = 148 animals) in the Campania region, Italy.

	Sample Size	No. of Infected Boars	Prevalence	SE	CI 95% *
Total	148	110	74.32%	7.04	67.29–81.36
PCV-2		70	47.30%	8.04	39.25–55.34
PCV-3		73	49.32%	8.05	41.27–57.38
PCV-2/3		33	22.30%	6.71	15.59–29.0

* Confidence intervals, 95%.

**Table 2:** Molecular prevalence of infection with PCV-2 or PCV-3, and risk factor analysis by gender and location of wild boars in the Campania region as detected by real-time PCR.

Factor	*n*	Positive	%	SE % ^§^	CI 95% *	χ^2^	P	OR ^#^	CI 95% *
Total	148	110	74.32	7.04	67.29–81.36				
Gender									
Female	62	57	91.94	6.78	85.16–98.71				
						18.466	<0.0001	8.29	2.98–23.02
Male	76	44	57.89	11.1	46.79–69.0				
n/a	10	9	90.0	18.59	71.4–100.0				
Province									
Caserta	32	29	90.6	10.1	80.53–100.0			Ref ^$^	
Avellino	30	20	66.6	16.87	49.8–83.54			4.83	1.17–19.8
Benevento	12	9	75.0	24.5	50.5–99.5	6.018	0.1108	3.22	0.55–18.84
Salerno	74	52	70.27	0.104	59.86–80.68			4.09	1.12–14.8

^§^ Standard error; * confidence intervals, 95%; ^#^ odds ratio; ^$^ reference category.

**Table 3:** Numbers of tissue sample tested and their percentages of positives to porcine circoviruses.

Sample	Sample Size	N. Infected Samples	Circoviruses Positivity (%) (PCV-2/PCV-3)	95% CI *	PCV-2 (%)	PCV-3 (%)	Coinfected (%)
Brain	104	60	57.7	48.20–67.19	20.2	20.2	17.3
Heart	119	67	56.3	47.39–65.21	16.8	21.8	17.6
Liver	136	89	65.4	57.45–73.43	22.1	21.3	22.1
Spleen	126	91	72.2	64.4–80.04	23.0	24.6	24.6

* Confidence intervals, 95%.

**Table 4:** Average number of PCV-2 or PCV-3 genomes (copies/µL) found in the different wild boar organs, analyzed by real-time quantitative PCR.

Sample	PCV-2 Average Quantity (Copies/µL)	PCV-3 Average Quantity (Copies/µL)
Brain	4.98 × 10^3^	1 × 10^1^
Heart	7.66 × 10^3^	1.5 × 10^1^
Liver	1.69 × 10^4^	2.0 × 10^1^
Spleen	4.98 × 10^6^	2 × 10^3^
Lymphnode	4.71 × 10^3^ *	2.96 × 10^3^ *

* Lymphonode results (even though referring to one sample only) were added, since these organs are usually employed to quantify PCV2 genomic copies for diagnostic purposes.
